# Docking, Synthesis and Evaluation of the Antifungal Activity of Pyrimido [4,5-b]quinolins 

**DOI:** 10.22037/ijpr.2020.1101010

**Published:** 2020

**Authors:** Reza Araghi, Bi Bi Fatemeh Mirjalili, Leila Zamani, Soghra Khabnadideh, Kamiar Zomoridian, Zeinab Faghih, Hamidreza Arabi

**Affiliations:** a *Department of Chemistry, College of Science, Yazd University, Yazd, Iran. *; b *Pharmaceutical Sciences Research Center, Shiraz University of Medical Sciences, Shiraz,.Iran. *; c *Center of Basic Researches in Infectious Diseases, Department of Medical Mycology and Parasitology, School of Medicine, Shiraz University of Medical Sciences, Shiraz,.Iran.*

**Keywords:** Pyrimido[4, 5-*b*]quinoline, Antifungal activity, Heterogeneous solid acid, Fe_3_O_4_@SiO_2_-SnCl_4_, Molecular modeling

## Abstract

In order to expand the application of Fe_3_O_4_@SiO_2_-SnCl_4_ in the synthesis of heterocyclic compounds, in this study, we wish to report the use of one-pot three component synthesis of pyrimido [4,5-*b*]quinolone derivatives (***D1-D16***) through reaction of 6-amino-2-(methylthio)pyrimidin-4 (3*H*)-one, dimedone, or 1,3-cyclohexadione and aldehydes in the presence of Fe_3_O_4_@SiO_2_-SnCl_4_ as an efficient eco-friendly catalyst under ultrasound irradiation. The final aim of this study is evaluation of antifungal activity of resulted products.

Synthesis of pyrimido [4,5-*b*]quinolin derivatives were done *via* three components coupling reaction of aldehyde, dimedone or 1,3-cyclohexadione and 6-amino-2-(methylthio)pyrimidin-4 (3*H*)-one in the presence of Fe_3_O_4_@SiO_2_-SnCl_4_ under ultrasonic irradiation in water at 60 °C. The products structure were studied by FT-IR^I^, ^1^H NMR,^II^ and ^13^C NMR^II^. All the compounds were screened for antimicrobial activity by broth microdilution methods as recommended by CLSI^III^. Considering our results showed that compound (***D13***) had the most antifungal activity against *C. dubliniensis*, *C. Albicans*, *C. Tropicalis,* and *C. Neoformance* at concentrations ranging (MIC90) from 1-4 μg/mL. Compounds (***D9***), (***D10***), (***D14***), and (***D15***) had significant inhibitory activities against *C. dubliniensis* at concentrations ranging (MIC90) from 4-8 μg/mL, respectively. 5-(3,4-dihydroxyphenyl)-8,8-dimethyl-2-(methylthio)-5,8,9,10-tetrahydropyrimido[4,5-*b*]quinoline-4,6(3*H*,7*H*)-dione (***D13***) exhibited inhibitory and fungicidal activities against the tested yeasts. The specific binding mode or the binding orientation of more efficient compounds to CYP51 active site, have been also performed by molecular modeling investigations and showed that there is a good correlation with biological test.

## Introduction

Pyrimidines are an important class of heterocyclic compounds present in various natural products and as scaffolds in numerous pharmaceutically important molecules and functional materials (1). Pyrimidoquinoline derivatives have been used in a number of biologically active compounds with anticancer (2), antimicrobial (3), antimalarial (4), and anti- inflammatory activities (5).

Few methods for the synthesis of pyrimido quinoline derivatives have been reported using MCRs in the presence of 1-*n*-butyl-3-methylimidazolium bromide ([bmim]Br) (6), *p*-toluenesulfonic acid (*p*-TSA) (7), indium(III) chloride (InCl_3_) (8), magnetic nanoparticles supported silica sulfuric acid (Fe_3_O_4_@SiO_2_-SO_3_H) (9), cellulose sulfuric acid (10), sulfonic acid supported on hydroxyapatite-encapsulated *γ*-Fe_2_O_3_ [*γ*-Fe_2_O_3_@HAp-SO_3_H] (11), nano-Fe_3_O_4_@cellulose-SO_3_H (12), Fe_3_O_4 _nano-particles supported on cellulose (Fe_3_O_4 _NPs-cell) under ultrasonic irradiation (13) and under microwave irradiation (14). Owing to the wide applications and significance of pyrimido quinoline derivatives in organic synthesis, pharmacology, and industry, there is still the need to develop an efficient, green, mild, and environmentally benign protocol for the synthesis of these important compounds.

 We have recently reported efficient and eco-friendly procedures for the preparation of xanthene and 1,4-dihydropyridine derivatives, using Fe_3_O_4_@SiO_2_-SnCl_4_ (15, 16) under ultrasound irradiation. In order to expand the application of Fe_3_O_4_@SiO_2_-SnCl_4_ in the synthesis of heterocyclic compounds, in this study, we wish to report the use of one-pot three component synthesis of pyrimido[4,5-*b*]quinolone derivatives through reaction of 6-amino-2-(methylthio)pyrimidin-4(3*H*)-one, dimedone, or 1,3-cyclohexadione and aldehydes in the presence of Fe_3_O_4_@SiO_2_-SnCl_4_ as an efficient eco-friendly catalyst under ultrasound irradiation. Also, some of the synthesized compounds were evaluated for their antimicrobial activity. In addition, docking simulation was performed to show the binding mode of the most efficient compounds to CYP51 active site.

## Experimental

*Material and methods*

Chemicals and solvents were purchased from Merck and Aldrich companies. FT-IR spectra were recorded as KBr pellet on a Bruker, Equinox 55 spectrometer. ^1^H NMR and ^13^C NMR spectra were recorded on a 400 MHz Bruker DRX-400 in DMSO-d_6_ as solvent and TMS as an internal standard. Melting points were obtained with a Buchi melting point B-540 B.V.CHI apparatus. Ultrasonic irradiations were done using Elmasonic S 40H ultrasonic cleaning.

*General procedure for the synthesis of pyrimido [4,5-b] quinolones*

In a round-bottom flask, a mixture of 6-amino-2-(methylthio)pyrimidin-4(3*H*)-one (1 mmol), aldehydes (1 mmol), dimedone or 1,3-cyclohexadione (1 mmol), and Fe_3_O_4_@SiO_2_-SnCl_4_ (0.03 g) in distilled H_2_O was sonicated in an ultrasonic cleaning unit (Elmasonic S 40H) at 60 °C for the stipulated time mentioned in table 2. The progress of the reaction was monitored by TLC (EtOAc:petroleum ether 7:3). After completion of the reaction, the catalyst was separated by an external magnet and reused for the next experiment. The reaction mixture was cooled to room temperature and then poured in to cold water. The solid product was filtered and washed with boiling water and recrystallized from ethanol to give the pure product in excellent yield.

*Determination of antifungal activities*

*Microorganisms*

The antifungal activities of the synthetic compounds against some American Type Culture Collection (ATCC) strains of fungi, including *Aspergillus flavus* (ATCC 64025), *Aspergillus* *fumigatus* (ATCC 14110), *Aspergillus* *clavatus*, *Staphylococcus* aureus, Candida albicans (ATCC 1912), *Candida albicans* (ATCC 1905), *Candida albicans* (SUCC 2303), *Candida albicans* (SUCC 625), *Candida glabarata* (ATCC 2192), *Candida glabarata* (ATCC 863), *Candida glabarata* (ATCC 2175), *Candida dubliniensis* (ATCC 7988), *Candida tropicalis* (SUCC 194), *Candida tropicalis* (SUCC 611), *Candida tropicalis* (ATCC 750), *C. Parapsilosis, Cryptococcus* *neoformance* (ATCC 9011), *Exophilia*, as well as two clinical isolates of yeasts identified by PCR-RFLP were determined. The susceptibility of all clinical isolates of fungi against selected antibiotics was examined by microdilution and disk diffusion methods (17).

*Determination of minimum inhibitory concentration*

MICs were determined using the broth microdilution method recommended by the CLSI with some modifications. Briefly, for determination of antimicrobial activities against fungi, serial dilutions of the synthetic compounds (1–1024 μg/mL) were prepared in 96-well microtiter plates using RPMI-1640 media (Sigma, St. Louis, MO, USA) buffered with MOPS (Sigma). Stock inoculums were prepared by suspending three colonies of the examined yeast in 5 mL sterile 0.85% NaCl, and adjusting the turbidity of the inoculums to 0.5 McFarland standards at 530 nm wavelengths (this yields stock suspension of 1-5 × 106 cells/mL). For moulds (Aspergillus spp. and Dermatophytes), conidia were recovered from the 7- day old cultures grown on potato dextrose agar by a wetting loop with tween-20. The collected conidia were transferred in sterile saline and their turbidity was adjusted to OD = 0.09-0.11 that yields 0.4-5 × 106 conidia/mL. Working suspension was prepared by making a 1/50 and 1/1000 dilution with RPMI of the stock suspension for moulds and yeasts, respectively. Working inoculums (0.1 mL) were added to the microtiter plates, which were incubated in a humid atmosphere at 30 ºC for 24–48 h. Uninoculated medium (200 μL) was included as a sterility control (blank). In addition, growth controls (medium with inoculums but without antibiotics or the synthetic compounds) were also included. The growth in each well was compared with that of the growth in the control well.

*Docking procedure*

An in house batch script (DOCK-FACE) for automatic running of Auto Dock 4.2 was used to carrying out the docking simulations in a parallel mode, using all system resources as described before. To prepare the receptor structure, the complex of Mycobacterium tuberculosis-CYP51 enzyme with Fluconazole (PDB ID: 1EA1) was acquired from Protein Data Bank (PDB data base; http://www.rcsb.org) and water molecules and co-crystal ligand were removed from the structure. The PDB were then checked for missing atom types with MODELLER 9.17.

The ligand structures were made by using HyperChem software package (Version 7, Hypercube Inc). For geometry optimization, Molecular Mechanic (MM^+^), followed by semi empirical AM1 method was performed. The prepared ligands were given to 100 independent genetic algorithm (GA) runs. 150 population size, a maximum number of 2,500,000 energy evaluations and 27,000 maximum generations were used for Lamarckian GA method. The grid points of 40, 40, and 40 in x-, y-, and z directions were used. A grid spacing of 0.375 A° was built centered on Hem group in the catalytic site of the receptor. Number of points in x, y, and z was -18, -3, and 67, respectively. All visualization of protein ligand interaction was evaluated using VMD software.

## Results and Discussion

*Synthesis*

**Scheme 1:**
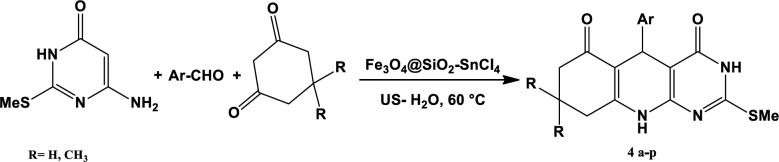
Synthesis of pyrimido[4,5-*b*]quinolones in the presence of Fe_3_O_4_@SiO_2_

Fe_3_O_4_@SiO_2_-SnCl_4_ as an efficient nano magnetic catalyst is a readily available catalyst. This catalyst is removed from the reaction medium by an external magnet. In our ongoing research for new synthetic methods for the development of efficient and environmentally friendly protocols for the synthesis of biologically important heterocyclic products, Pyrimido [4,5-*b*]quinolin derivatives were synthesized. These heterocyclic compounds were synthesized by 6-amino-2-(methylthio)pyrimidin-4(3*H*)-one (1), aryl aldehydes (2), dimedone or 1,3-cyclohexadione (3) and H_2_O (5 mL) in the presence of a nanocatalytic of Fe_3_O_4_@SiO_2_-SnCl_4_ under ultrasound irradiation (Scheme 1 and Table 1). The products were characterized by their melting points using FT-IR, ^1^H, and ^13^C NMR spectroscopy. 

The reusability of Fe_3_O_4_@SiO_2_-SnCl_4_ was examined for four times without any considerable decrease in its efficiency. 

**Scheme 2:**
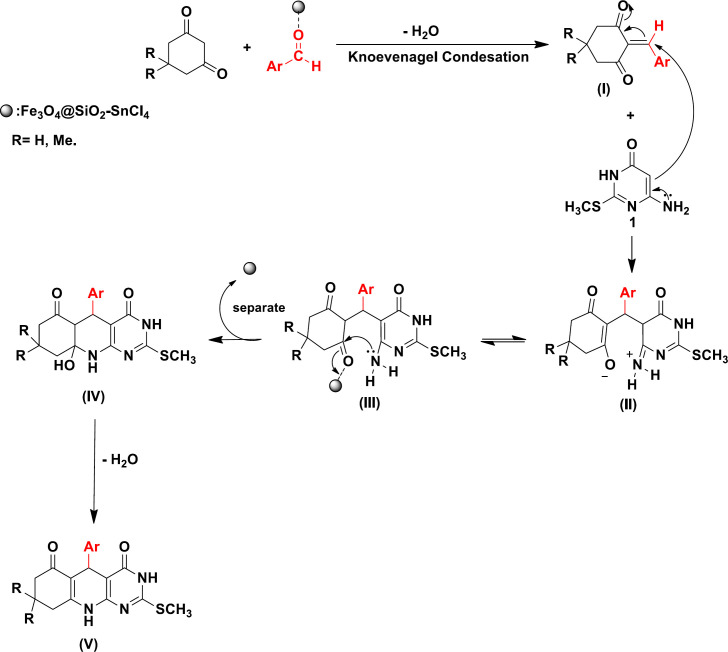
A proposed mechanism for preparation of pyrimido [4,5-*b*]quinolones in the presence of Fe_3_O_4_@SiO_2_-SnCl_4_

The mechanism of the reaction and formation of compound 4a can be explained by the condensation, addition, cyclization, and dehydration reactions. The plausible mechanism for the synthesis of pyrimido [4,5-*b*]quinolones in the presence of Fe_3_O_4_@SiO_2_-SnCl_4_, which can act as Lewis acid catalyst is depicted in scheme 2.

The carbonyl oxygen of aldehyde coordinates with the Lewis acid moiety increasing the electrophilicity of the carbonyl carbon and thereby making it possible to carry out the reaction in short time. In a plausible mechanism, it is assumed that the reaction may proceed initially through the Knoevenagel condensation between aryl aldehydes and dimedone or 1,3-cyclohexadione to form intermediate (I). Next, Michael addition of 6-aminouracil to intermediate (I) affords (II). Intermediate (II) converts to (III) after tautomerization (Fe_3_O_4_@SiO_2_-SnCl_4_ can also act as a mild base for the deprotonation of an acidic proton of Intermediate (II). Then, intermediate (III) converts to (IV) *via* cyclization. Finally, the desired product (V) is obtained after dehydration of (IV) (Scheme 2).

*Antifungal activities of the synthetic compounds*

In this study, compounds (***D1-D16***) evaluated against fungi (Table 2). None of the compounds have any effect on bacteria. Considering our results showed that compound (***D13***) had the most antifungal activity against *C. dubliniensis,* *C. Albicans*, *C. Tropicalis,* and *C. Neoformance* at concentrations ranging (MIC90) from 1-4 μg/mL. Compounds (***D9***), (***D10***), (***D14***), and (***D15***) had significant inhibitory activities against *C. dubliniensis* at concentrations ranging (MIC90) from 4-8 μg/mL, respectively. In comparison of the antifungal activities of the synthetic compounds based on variation of substitutions on 2,3, and 4-position of phenyl ring, we found that the compound ***D13*** with OH residue in meta and para positions of phenyl ring exhibited a better antifungal activities against the tested fungi than the other compounds. Compounds (***D9***) and (***D10***), had significant inhibitory activities against *C. Dubliniensis*, respectively. we found that the compound (***D9***) with Br residue in para position of phenyl ring exhibited a better antifungal activity against the *C. Dubliniensis *than the compound (***D10***) with CH_3_ residue in para position of phenyl ring.

**Figure 1:**
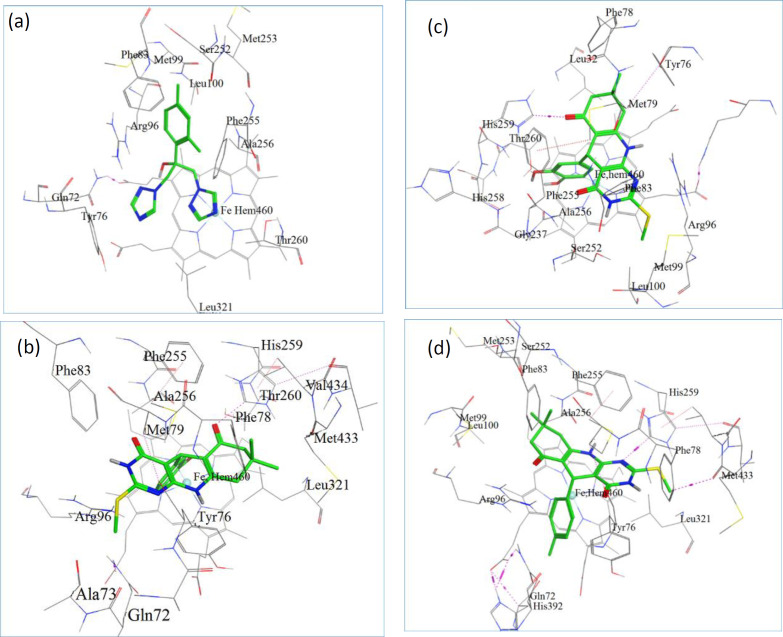
(a) The docked configuration of Fluconazole in the binding site of lanosterol 14α-demethylase (CYP51); (b) the docked configuration of D9; (c) the docked configuration of D10; and (d) the docked configuration of D13 in the binding site. Hydrogen bonds are shown as purple dotted lines

**Table 1.** Synthesis of pyrimido [4,5-*b*]quinolone derivatives under ultrasound irradiation

Yield (%)^a^	Time (min)	Product	R	Ar	Entry
98	75	4l	Me	4-OMeC_6_H_4_	**D1**
97	80	4m	Me	3,4-OMe_2_C_6_H_3_	**D2**
99	55	4c	Me	2,4-Cl_2_C_6_H_3_	**D3**
99	60	4b	Me	4-NO_2_C_6_H_4_	**D4**
96	90	4j	Me	C_6_H_5_	**D5**
97	75	4i	Me	4-OH-3-OMeC_6_H_3_	**D6**
98	65	4g	Me	4-CO_2_MeC_6_H_4_	**D7**
99	50	4d	Me	3,4,5-F_3_C_6_H_2_	**D8**
98	65	4e	Me	3-BrC_6_H_4_	**D9**
97	80	4k	Me	4-MeC_6_H_4_	**D10**
99	60	4a	Me	4-ClC_6_H_4_	**D11**
98	60	4f	Me	3-NO_2_C_6_H_4_	**D12**
97	80	4h	Me	3,4-OH_2_C_6_H_3_	**D13**
99	70	4p	H	4-NMe_2_C_6_H_4_	**D14**
98	65	4o	H	4-CO_2_MeC_6_H_4_	**D15**
98	60	4n	H	2-ClC_6_H_4_	**D16**

**Table 2:** Minimum inhibitory and fungicidal concentrations of the synthetic compounds (µg/mL) against the examined fungi

*Compounds*																*concentration*	*Fungi*
***D16***	***D15***	***D14***	***D13***	***D12***	***D11***	***D10***	***D9***	***D8***	***D7***	***D6***	***D5***	***D4***	***D3***	***D2***	***D1***		
-	-	-	4	-	-	-	-	-	-	-	-	-	-	-	-	MIC90	*C.albicans*
-	-	-	16	-	-	-	-	-	-	-	-	-	-	-	-	MFC	
-	-	-	-	-	-	-	-	-	-	-	-	-	-	-	-	MIC90	*C.glabrata*
-	-	-	-	-	-	-	-	-	-	-	-	-	-	-	-	MFC	
-	-	-	-	-	-	-	-	-	-	-	-	-	-	-	-	MIC90	*C.krusei*
-	-	-	-	-	-	-	-	-	-	-	-	-	-	-	-	MFC	
-	-	-	1	-	-	-	-	-	-	-	-	-	-	-	-	MIC90	*C.tropicalis*
-	-	-	G	-	-	-	-	-	-	-	-	-	-	-	-	MFC	
-	-	-	-	-	-	-	-	-	-	-	-	-	-	-	-	MIC90	*C.parapsilosis*
-	-	-	-	-	-	-	-	-	-	-	-	-	-	-	-	MFC	
-	-	-	-	-	-	-	-	-	-	-	-	-	-	-	-	MIC90	*Exophilia*
-	-	-	-	-	-	-	-	-	-	-	-	-	-	-	-	MFC	
-	-	-	-	-	-	-	-	-	-	-	-	-	-	-	-	MIC90	*A.clavatus*
-	-	-	-	-	-	-	-	-	-	-	-	-	-	-	-	MFC	
-	-	-	-	-	-	-	-	-	-	-	-	-	-	-	-	MIC90	*A.fumigatus*
-	-	-	-	-	-	-	-	-	-	-	-	-	-	-	-	MFC	
-	-	-	-	-	-	-	-	-	-	-	-	-	-	-	-	MIC90	*A.flavus*
-	-	-	-	-	-	-	-	-	-	-	-	-	-	-	-	MFC	
-	-	-	2	-	-	-	-	-	-	-	-	-	-	-	-	MIC90	*C.neoformans*
-	-	-	2	-	-	-	-	-	-	-	-	-	-	-	-	MFC	
-	32	8	4	-	-	4	2	-	-	-	-	-	-	-	-	MIC90	*C.dubliniensis*
-	G	8	4	-	-	4	2	-	-	-	-	-	-	-	-	MFC	
-	-	-	-	-	-	-	-	-	-	-	-	-	-	-	-	MIC90	*S.aurous*
-	-	-	-	-	-	-	-	-	-	-	-	-	-	-	-	MFC	
-	-	-	-	-	-	-	-	-	-	-	-	-	-	-	-	MIC90	*E.coli*
-	-	-	-	-	-	-	-	-	-	-	-	-	-	-	-	MFC	
-	-	-	-	-	-	-	-	-	-	-	-	-	-	-	-	MIC90	*E.fecalis*

**Table 3:** Molecular docking Studies on the CYP51 active site

**Compound**	**Binding Energy (Kcal/mol)** ^*^
***D9***	-8.04
***D10***	-7.59
***D13***	-8.86
***Fluconazol***	-6.47

^ *^All the docking protocols were performed on validated structures with RMSD

values below 2 Å.

*Docking study of active compounds*

Docking is a method, frequently used to find the binding orientation of small molecule drug candidates to their protein targets in order to predict and interpret the affinity and activity of the small molecule. We performed molecular docking studies on ***D9****, ****D10****, ****D13,*** and **Fluconazole** to find, compare and validate their binding site, binding modes, and their best direction according to their binding energy (Figures 1). Once the docking procedure was completed, to find the type of interactions, the protein–ligand complex was studied. All the docking protocols were done on validated structures, with RMSD values below 2 Å. The conformation with the lowest ones was considered as the best docking result. Docking binding energies of these active compounds were summarized in Table 3. Our results indicated that overall there is a good correlation between experimental pIC50 and docking binding energy (Figure 1). As displayed in Table 3, all investigated complexes showed better docking binding energies than the co-crystal ligands (fluconazole)**. **Consistent with our biological results, ***D13*** was observed to have the best docking binding energies with the receptor through polar interaction with phe 255, His 259, and Thr 260 which responds to the key ineractions of pharmacophoric elements in the ligands (Figures 1). 

The docking model also indicated the specific alignment of fused three ring system to the pocket of heme iron of CYP51 in each ligand with similar positioning of triazole ring of fluconazole. This orientation with respect to the hydrogen bonding and hydrophobic interactions may be in favor of antifungal activity.

## Conclusion

In conclusion, a convenient and efficient method for the synthesis of pyrimido[4,5-*b*]quinolin derivatives in the presence of Fe_3_O_4_@SiO_2_-SnCl_4_ as a novel heterogeneous solid acid catalyst has been developed through ultrasound irradiation. This reaction under ultrasound irradiation not only gave excellent yield of products with lesser reaction time but it also required mild reaction conditions and exhibited operational simplicity. In addition, low cost, availability, recyclability, low toxicity of the catalyst and the use of water as a green reaction medium make this methodology a valid contribution to the existing processes in the field of one-pot multicomponent reaction. 

Considering our results showed that compound 5-(3,4-dihydroxyphenyl)-8,8-dimethyl-2-(methylthio)-5,8,9,10 tetrahydropyrimido[4,5-*b*]quinoline-4,6(3*H*,7*H*)-dione (***D13***) had the most antifungal activity against *C. dubliniensis,* *C. Albicans*, *C. Tropicalis* and *C. Neoformance* at concentrations ranging (MIC90) from 1-4 μg/mL. Compounds (***D9***), (***D10***), (***D14***), and (***D15***) had significant inhibitory activities against *C. dubliniensis* at concentrations ranging (MIC90) from 4-8 μg/mL, respectively. In line with our biological data, ***D13*** had the highest docking binding energy, as well it indicated that there is a good correlation between the docking studies and the antifungal activity results. (***D9***), (***D10****)*, (***D14****),* and (***D15***) have medium antifungal activity but the other compounds do not have any antifungal activity. 
